# The Future of Seed Amplification Assays and Clinical Trials

**DOI:** 10.3389/fnagi.2022.872629

**Published:** 2022-06-22

**Authors:** Thomas Coysh, Simon Mead

**Affiliations:** ^1^MRC Prion Unit at UCL, UCL Institute of Prion Diseases, London, United Kingdom; ^2^National Prion Clinic, National Hospital for Neurology and Neurosurgery, University College London Hospitals NHS Foundation Trust, London, United Kingdom

**Keywords:** RT-QuIC, protein aggregation, prion, neurodegenerative diseases, seed amplification assays, biomarker, pre-symptomatic diagnosis, pre-symptomatic carriers

## Abstract

Prion-like seeded misfolding of host proteins is the leading hypothesised cause of neurodegenerative diseases. The exploitation of the mechanism in the protein misfolding cyclic amplification (PMCA) and real-time quaking-induced conversion (RT-QuIC) assays have transformed prion disease research and diagnosis and have steadily become more widely used for research into other neurodegenerative disorders. Clinical trials in adult neurodegenerative diseases have been expensive, slow, and disappointing in terms of clinical benefits. There are various possible factors contributing to the failure to identify disease-modifying treatments for adult neurodegenerative diseases, some of which include: limited accuracy of antemortem clinical diagnosis resulting in the inclusion of patients with the “incorrect” pathology for the therapeutic; the role of co-pathologies in neurodegeneration rendering treatments targeting one pathology alone ineffective; treatment of the primary neurodegenerative process too late, after irreversible secondary processes of neurodegeneration have become established or neuronal loss is already extensive; and preclinical models used to develop treatments not accurately representing human disease. The use of seed amplification assays in clinical trials offers an opportunity to tackle these problems by sensitively detecting *in vivo* the proteopathic seeds thought to be central to the biology of neurodegenerative diseases, enabling improved diagnostic accuracy of the main pathology and co-pathologies, and very early intervention, particularly in patients at risk of monogenic forms of neurodegeneration. The possibility of quantifying proteopathic seed load, and its reduction by treatments, is an attractive pharmacodynamic biomarker in the preclinical and early clinical stages of drug development. Here we review some potential applications of seed amplification assays in clinical trials.

## Introduction

Misfolding, aggregation, and accumulation of specific host proteins within or outside cells is common to many neurodegenerative diseases (Soto and Pritzkow, 2018). Proteinopathies of β-amyloid, tau, α-synuclein, TAR DNA-binding protein 43 (TDP-43), fused in sarcoma (FUS), and prion protein account for the vast majority of human neurodegenerative diseases (Kovacs, 2016). Increasing evidence implicates so-called prion-like seeded protein misfolding and aggregation as a central underlying mechanism (Prusiner et al., 2015; Collinge, 2016; Soto and Pritzkow, 2018). Whilst “prion-like” is a commonly used descriptor of a disease model, more important are the implications of the model. Seed amplification assays have been transformational in prion disease research and diagnostics, and are increasingly being used in research into other neurodegenerative diseases. Their ability to sensitively detect and quantify *proteopathic seeds*, self-propagating misfolded host protein assemblies which seed the polymerisation, and templated misfolding of normal host proteins, represents a potentially powerful tool. Applications might include *antemortem* diagnosis of neurodegenerative disease, identification of co-pathologies, and assessment of drug target engagement.

In an era of therapeutic development targeting the pathobiology underlying different neurodegenerative disorders, accurate *antemortem* diagnosis is of great importance. It will enrich clinical trials with patients who have the pathology that the experimental treatment is designed to modify, increasing the chances of detecting effective treatments and reducing futile exposures to drugs. Furthermore, through sensitive detection of disease-specific pathology, seed amplification assays could facilitate pre-symptomatic diagnosis and trials of therapeutics prior to clinical presentation, when the neuronal loss is less extensive and successful treatment is anticipated to be more likely. Panels of seed amplification assays for different proteopathic seeds could enhance antemortem detection of co-pathologies and the understanding of their contribution to disease phenotype and progression and could guide the rational selection of agents in trials of combination therapies (Kovacs, 2016). Quantitative seed amplification assays also offer the possibility of measuring the effects of therapeutics in reducing proteopathic seed load, an attractive pharmacodynamic biomarker (Wilham et al., 2010; Shi et al., 2015).

Seed amplification assays can identify the biologically relevant aggregation-inducing proteopathic seeds antemortem, using amplification of minute amounts of seeds from accessible biofluids or tissues (Saborio et al., 2001; Wilham et al., 2010; Atarashi et al., 2011; Bongianni et al., 2017), in contrast to many other fluid biomarkers of neurodegeneration such as 14-3-3 and neurofilament light chain which are secondary markers of neural damage rather than underlying proteinopathy. Here, Alzheimer’s disease is a notable exception, in which measurement of reduced β-amyloid 1-42 monomer is thought to reflect its equilibrium with amyloid deposited in the brain (Simrén et al., 2020).

**Figure 1:**
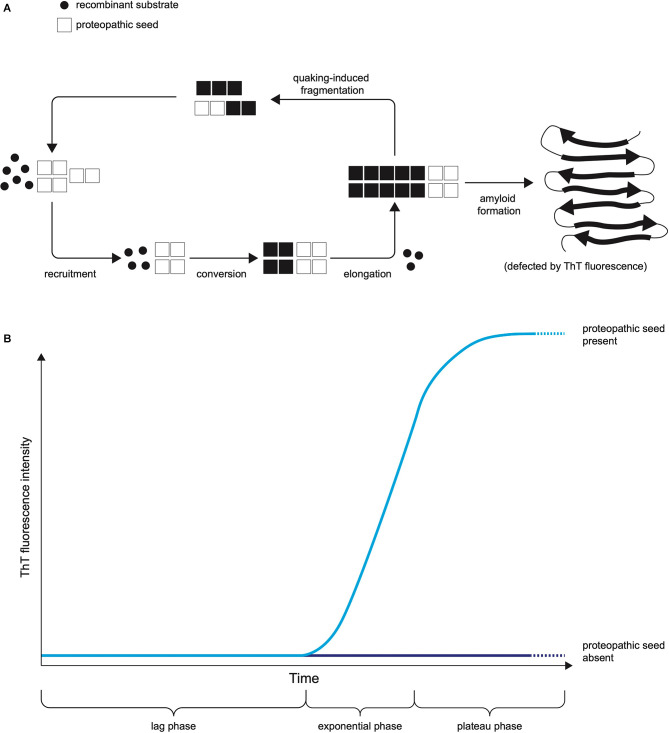
The Principle of RT-QuIC. **(A)** The mechanistic principle of proteopathic seed amplification using multiple cycles of incubation and quaking-induced fragmentation resulting in beta-sheet rich amyloid formation. **(B)** Schematic representation of RT-QuIC readout due to ThT fluorescence as protein amyloid accumulates. The kinetics of the RT-QuIC readout (rate of rise, maximum fluorescence, lag phase) may vary according to the type of proteopathic seed and its quantity, as well as assay conditions including temperature, shaking parameters, the composition of reaction mix, and choice of substrate.

The two leading types of seed amplification assay, developed in the field of prion disease, are the real-time quaking-induced conversion (RT-QuIC; Wilham et al., 2010), summarised in Figure 1, and the protein misfolding by cyclic amplification (PMCA; Saborio et al., 2001) assays. Both harness the ability of normal prion protein substrate to be converted into misfolded aggregates by proteopathic seeds of misfolded prion protein and utilise kinetic energy to repeatedly break down the nascent aggregates, amplifying proteopathic seeds and accelerating the conversion of normal substrate to misfolded product. In RT-QuIC the kinetic energy source is physical shaking (quaking), whereas in PMCA repeated cycles of sonication are used. In RT-QuIC the substrate for templated misfolding is recombinant prion protein whereas in classical PMCA it is normal brain homogenate or another biological source of prion protein. In RT-QuIC the products of the reaction are non-infectious amyloids whereas PMCA produces infectious prions with the fidelity of the prion strain (prion strains are proposed to be conformational variants of prions that are associated with clinicopathological phenotypes and are transmissible between hosts). The readout in RT-QuIC is Thioflavin T (ThT) fluorescence, which increases as the substrate is converted to amyloid, whereas in PMCA it is an immunoblot or immunoassay (Saborio et al., 2001). The RT-QuIC, despite losing some biological information by not reproducing infectious prion strains, has practical features advantageous for use in a clinical laboratory including non-infectious reaction products (Zanusso et al., 2016), ThT fluorescence providing a readout easily measured in real-time (compared to labour and time-intensive Western blotting) and recombinant protein as substrate rather than brain homogenate (Zanusso et al., 2016).

Improvements in RT-QuIC sensitivity and specificity for sporadic Creutzfeldt-Jakob disease (sCJD) have been brought about by iterative changes in assay conditions including temperature, shaking parameters, and the composition of reaction mix and choice of substrate (Orrú et al., 2016; Metrick et al., 2019). First generation RT-QuIC, also known as PQ-CSF RT-QuIC, which uses full length 23-231 recombinant hamster prion protein (rPrP) as substrate provides excellent specificity (98%–100%; Atarashi et al., 2011; McGuire et al., 2012; Bongianni et al., 2017; Lattanzio et al., 2017) but variable sensitivity with poorer sensitivity for atypical forms of sCJD (MV2K, MM2C, and MM2T) than Parchi MM1 (Lattanzio et al., 2017; Abu-Rumeileh et al., 2019). The second generation or IQ-CSF RT-QuIC, which uses truncated hamster rPrP 90-231 as a substrate, addition of sodium dodecyl sulphate to the reaction mix and an increased temperature, has superior sensitivity and shorter run time without compromising on specificity for sCJD, as confirmed by multiple groups (Orrú et al., 2015a; Bongianni et al., 2017; Foutz et al., 2017; Franceschini et al., 2017; Groveman et al., 2017; Fiorini et al., 2020; Rhoads et al., 2020). Studies comparing first and second generation RT-QuIC on the same patients’ samples have confirmed superior sensitivity of the second generation RT-QuIC (Franceschini et al., 2017; Groveman et al., 2017). In sCJD the RT-QuIC has been incorporated into diagnostic criteria (European Centre for Disease Prevention and Control, 2017) owing to its reproducible sensitive and specific detection of proteopathic seeds in sCJD, with ring trials of first and second-generation RT-QuIC now demonstrating reproducibility between different laboratories internationally (McGuire et al., 2016; Orrú et al., 2020).

The principles of RT-QuIC developed in prion disease diagnosis have subsequently been applied to biosamples in a range of neurodegenerative diseases with proposed proteopathic seeds including those composed of β-amyloid, α-synuclein, tau, and TDP-43, summarised in Table 1. In future, they may also be applied to other proteopathic disorders with seeded protein misfolding and aggregation. There has been some evolution in terminology with the Soto group’s work on α-synuclein PMCA adhering more to the principles of RT-QuIC than classical PMCA described above (Shahnawaz et al., 2020), and a recent suggestion for seed amplification assay to be used as an umbrella term to encompass both techniques (Russo et al., 2021). For simplicity, we will use the terminology defined here in this review. We anticipate wider adoption of seed amplification assays for proteopathic seeds into diagnostic criteria for neurodegenerative diseases as the technology develops. It will remain important for the sensitivity and specificity of such tests to be confirmed in multiple centres with longitudinal studies and, wherever possible, with neuropathological confirmation of diagnosis.

**Table 1:** Summary of RT-QuIC assays for proteopathic seeds in biosamples^a^.

Protein seed	Disease	Analyte	Sensitivity	Specificity	References/Notes
PrP	Sporadic CJD	CSF	73%–100%	98%–100%	First generation RT-QuIC (Atarashi et al., 2011; McGuire et al., 2012; Bongianni et al., 2017; Lattanzio et al., 2017) less sensitive than second generation RT-QuIC (Orrú et al., 2015a; Bongianni et al., 2017; Foutz et al., 2017; Franceschini et al., 2017; Groveman et al., 2017; Fiorini et al., 2020; Rhoads et al., 2020). First generation RT-QuIC is less sensitive in atypical strains (Parchi MV2K, MM2C and MM2T) than Parchi MM1 (Lattanzio et al., 2017; Abu-Rumeileh et al., 2019).
		Nasal brushings	90%–97%	100%	Orrú et al. (2014), Bongianni et al. (2017), and Fiorini et al. (2020). In combination with CSF RT-QuIC sensitivity approaches 100% (Bongianni et al., 2017; Fiorini et al., 2020).
		Skin	89%–100%	86%–100%	The preparation of skin samples varied between studies (Orrú et al., 2017; Mammana et al., 2020; Xiao et al., 2021).
		Eye	11/11 sCJD cases positive	6/6 controls negative	This study did not set out to define sensitivity and specificity but simply to demonstrate seeding activity in the eyes of sCJD patients (Orrú et al., 2018)
	Inherited prion diseases	CSF & nasal brushings	0%–100%	98%–100%	Variable sensitivity and specificity reported in IPD due to different mutations, with performance better in IPD with a CJD phenotype (e.g., E200K) than slowly progressive IPD (e.g., P102L) (Cramm et al., 2015; Bongianni et al., 2017; Foutz et al., 2017; Franceschini et al., 2017; Lattanzio et al., 2017; Rhoads et al., 2020). One report of good diagnostic accuracy in slow IPD with RT-QuIC using recombinant human PrP substrate (Sano et al., 2013).
Alpha-synuclein	Parkinson’s disease	CSF	84%–100%	80%–100%	Fairfoul et al. (2016); Shahnawaz et al. (2017); Shahnawaz et al. (2020); Groveman et al. (2018); Garrido et al. (2019); Kang et al. (2019); Manne et al. (2019); van Rumund et al. (2019); Rossi et al. (2020); and Bargar et al. (2021b) Lower sensitivity (40%–78%) in LRRK2-associated PD, possibly due to non-α-synuclein pathologies underlying this syndrome and not useful in autosomal recessive forms of PD associated with α-synuclein-negative nigral degeneration (Garrido et al., 2019; Brockmann et al., 2021).
		Nasal brushings	46%–69%	83%–95%	De Luca et al. (2019); Bargar et al. (2021a); and Stefani et al. (2021). Lower sensitivity for PD than MSA-P.
		Skin	76.9%–100%	80%–100%	Manne et al. (2020b); Wang et al. (2020); Donadio et al. (2021); Kuzkina et al. (2021); and Mammana et al. (2021). Some of these studies used postmortem samples only, a combination of postmortem and intra-vitam samples and others intra-vitam samples only.
		Submandibular gland	100%	96%	Manne et al. (2020a). Single study using postmortem samples only.
		Saliva	76%	94%	Luan et al. (2022). Single study of clinically diagnosed patients and controls (no neuropathological conformation).
		Colon	N/A	N/A	Bargar et al. (2021b). This study demonstrated greater seeding activity in colonic tissue homogenate than CSF from a neuropathologically confirmed PD patient.
	Dementia with Lewy bodies	CSF	85%–100%	78%–100%	Fairfoul et al. (2016); Shahnawaz et al. (2017); Groveman et al. (2018); Bongianni et al. (2019); Rossi et al. (2020); and Bargar et al. (2021b). Includes studies with and without neuropathological confirmation.
		Skin	75%–100%	80%–96%	Donadio et al. (2021) and Mammana et al. (2021). Small numbers of DLB patients (3/4 and 17/17 DLB patients with positive seeding activity respectively).
	Multiple system atrophy	CSF	35%–85%	89%–98%	Shahnawaz et al. (2017); Shahnawaz et al. (2020); and van Rumund et al. (2019). Some studies found poor seeding activity in MSA (van Rumund et al., 2019; Rossi et al., 2020), but Shahnawaz et al. (2020) described lower fluorescence maxima of MSA compared to PD samples, and different kinetics, making discrimination possible.
		Nasal brushings	82% MSA 90% MSA-P 5% MSA-C	83%–95%	Bargar et al. (2021a) divided MSA into MSA-P and MSA-C and found differing sensitivities. De Luca et al. (2019) did not analyse according to MSA subtype.
		Skin	1/1 MSA-C 1/3 MSA-P 2/3 MSA	80%	Small studies with four and three MSA patients tested only (Wang et al., 2020; Donadio et al., 2021).
		Saliva	61%	94%	Single study with 18 MSA patients (Luan et al., 2022).
	Isolated REM sleep behaviour disorder	CSF	90%–100%	84%–98%	Rossi et al. (2020) and Iranzo et al. (2021). 97% of patients with iRBD who developed PD or DLB over follow-up of 7 years had positive CSF α-synuclein RT-QuIC.
		Nasal brushings	44%	90%	Stefani et al. (2021). Single cross-sectional study with 61 iRBD cases and 59 matched controls.
	Pure autonomic failure	CSF	93%	84%–98%	Rossi et al. (2020). In a different study aiming to distinguish PD and atypical Parkinsonian disorders, RT-QuIC was negative in all PAF patients (Quadalti et al., 2021).
		Skin	67%	80%	Donadio et al. (2021). Small single study with only three PAF patients.
Tau (3R)	Pick’s disease	CSF	91%–100%	94%–100%	Saijo et al. (2017). Single small study with post-mortem Pick’s disease CSF samples and antemortem control samples. Other studies detecting 3R tau seeds have used brain homogenate samples only (Kraus et al., 2019; Metrick et al., 2020; Tennant et al., 2020).
Tau (4R)	Progressive supranuclear palsy Corticobasal degeneration	CSF	Not done	Not done	In this study which established 4R Tau RT-QuIC, positive responses were obtained from all PSP and CBD post-mortem CSF samples and no controls but antemortem CSF samples had weaker seeding activity (Saijo et al., 2020). The other study detecting 4R tau seeds used brain homogenate samples only (Tennant et al., 2020).
Tau (3R/4R)	Alzheimer’s disease Chronic traumatic encephalopathy	Brain homogenate only	Not done	Not done	No CSF studies at present (Kraus et al., 2019; Metrick et al., 2020).
TDP-43	Amyotrophic lateral sclerosis Frontotemporal dementia	CSF	94%	85%	One report only with CSF samples from 35 patients with *C9orf72*, *GRN* and *TARDBP* mutations and one pre-symptomatic *GRN mutation* carrier (Scialò et al., 2020).
Amyloid-beta	Alzheimer’s disease	CSF	90%	92%	Salvadores et al. (2014). Only one study so far with limited numbers, other groups have found CSF inhibits amyloid-beta aggregation or that amyloid-beta has the propensity for spontaneous fibrillisation resulting in difficulty distinguishing true and false positives.

This review will focus on the application of these assays, particularly to clinical trials, and areas of expansion for RT-QuIC including TDP-43 and the potential for expansion into inherited disorders including inherited prion diseases, inherited Parkinson’s disease, and Huntington’s disease. Other reviews in this series focus on a detailed appraisal of the literature on PrP, tau, and α-synuclein seed amplification assays.

## Role of Protein Aggregation with Seed Amplification Assays in Clinical Trials

### Improving Accuracy of Ante-Mortem Diagnosis

Clinical features are a relatively poor predictor of pathology in many neurodegenerative disorders, and often require patients to be followed up over time for the requisite combination of diagnostic features to emerge, whereas a definite diagnosis is only made post-mortem. As an example, a post-mortem series of clinically diagnosed probable multiple system atrophy (MSA) revealed only 62% accuracy with other disorders including Parkinson’s disease (PD), Dementia with Lewy Bodies (DLB) and progressive supranuclear palsy (PSP) masquerading as MSA (Koga et al., 2015).

Molecular diagnosis with disease-specific seed amplification assays could enable trials of treatments targeting proteinopathies early in the disease course, prior to extensive irreversible neurodegeneration, whilst enriching for patients with the “correct” underlying pathobiology. Fluid biomarkers for Alzheimer’s disease (AD) in CSF and blood including different forms of β-amyloid and phosphorylated tau make such an approach possible in AD (Zetterberg and Blennow, 2018), although even these are indirect measures for the proteopathic seeds that are assumed to be driving neurodegeneration. The direct measurement of monomeric or oligomeric forms of other proteins such as α-synuclein as biomarkers has been challenging and has not yielded consistent results, whereas α-synuclein RT-QuIC permits sensitive and specific quantification of α-synuclein seeds in CSF (Fairfoul et al., 2016; Shahnawaz et al., 2017). This is preferable to tests currently used to support clinical diagnosis, which detect secondary effects of pathology, such as DaT scan identifying loss of striatal dopamine transporters, which occurs in α-synucleinopathies such as PD and MSA but also in PSP, a tauopathy, and corticobasal syndrome, associated with a range of pathologies.

Recently the utility of α-synuclein RT-QuIC as an early diagnostic biomarker has been demonstrated in patients with isolated rapid eye movement sleep behaviour disorder (iRBD), which can be a prodromal stage of α-synucleinopathies including DLB, PD, and MSA: 97% of patients with iRBD who went on to develop clinically overt PD or DLB over a follow-up of 7 years had positive CSF α-synuclein RT-QuIC (Iranzo et al., 2021). However, 10% of healthy controls who did not develop symptomatic α-synucleinopathy also exhibited α-synuclein seeding activity, raising the question of whether these represented false positives or pre-symptomatic α-synucleinopathies (Iranzo et al., 2021). Studies with quantitative biomarker outcomes, longer follow-up and neuropathological confirmation should help to answer these questions.

If an early or pre-symptomatic diagnosis with a view to therapeutic intervention is the aim, it will be essential to minimise false positive results and avoid detrimental impacts. There may be value in using orthogonal approaches to confirm that a neurodegenerative process is underway, such as biomarkers of neural damage, although evidence from prion disease in sheep suggests these will become positive later than RT-QuIC (Llorens et al., 2018). Equally, it will be important for more studies to be done to understand the implications of a true positive result in different disorders: for example, seeding activity might be present for years before a significant neuropathological change occurs, which may itself precede clinical disease by years in slowly progressive neurodegenerative diseases. On the other hand, data from inherited prion disease demonstrated positive RT-QuIC seeding activity in 1/10 asymptomatic carriers of the E200K *PRNP* mutation (which causes a rapid CJD-type phenotype), implying that seeding activity is not consistently present for long periods prior to symptom onset in E200K carriers, although the one seeding positive individual, who was near the median age of onset, remained asymptomatic 1 year later (Vallabh et al., 2020). Cohorts of asymptomatic individuals carrying mutations known to cause neurodegenerative disease can play a critical role in studies of temporal changes in seeding capacity as there is a greater likelihood of capturing a pre-symptomatic window and subsequent phenoconversion than in sporadic neurodegenerative diseases.

Combinations of seed amplification assays that identify the underlying proteinopathy together with other biomarkers, for example, neurofilament light chain as a measure of neuroaxonal breakdown, have the potential to further improve diagnostic accuracy. For example, in one recent study typical α-synuclein seeding activity on RT-QuIC distinguished PD from MSA, CBD, and PSP with a sensitivity of 91.4% and specificity 97.5%, whereas a combination of α-synuclein RT-QuIC positivity and NfL below a certain threshold achieved a sensitivity of 98.3% and specificity of 95.8% and a greater area under the receiver operating characteristic curve (Quadalti et al., 2021). With recent advances in ultrasensitive, high-throughput protein detection and commercial availability of plasma proteomics panels incorporating thousands of proteins, we anticipate progress in combinatorial approaches in which proteopathic seed identification could be combined with signatures of plasma protein alterations to improve diagnostic and prognostic classification, with potential for enrichment of clinical trials with patient subgroups most likely to benefit from specific treatments (Assarsson et al., 2014; Olink, 2021). The same combinatorial approach to improving diagnostic accuracy and precision applies to a combination of aggregation assays with microRNA or metabolomic signatures (Nagaraj et al., 2019; Shao and Le, 2019).

Different conformational strains of the same misfolded protein are thought to contribute to phenotypic differences in neurodegenerative diseases, such as the spectrum of prion diseases, tauopathies, or α-synucleinopathies (Parchi et al., 1999; Goedert et al., 2014; Peelaerts et al., 2015; Schweighauser et al., 2020; Shi et al., 2021). Structural characterisation of protein aggregates from patients’ brains using techniques such as cryo-electron microscopy has advanced significantly in recent years and may eventually become the gold standard for protein structure-based classification of these diseases (Fitzpatrick et al., 2017; Schweighauser et al., 2020; Shi et al., 2021). However, seed amplification assays detect the pathobiologically-relevant property of proteopathic seeding, and offer a more rapid, more affordable and higher-throughput assay, which provides the opportunity for molecular confirmation of proteopathic seed *in vivo* in accessible biofluids or tissues. RT-QuIC kinetics and maximal fluorescence intensity can potentially be used to indirectly discriminate different strains of the same protein, as demonstrated in prion disease (Peden et al., 2012; Foutz et al., 2017), although differences in RT-QuIC readouts between studies (and differences related to reaction conditions and recombinant substrates used) limit the practical application of such methods at present. More recently the Soto group demonstrated that their α-synuclein-PMCA (utilising the same principle as RT-QuIC; Shahnawaz et al., 2020; Singer et al., 2020) can discriminate between MSA and Lewy Body α-synucleinopathy seeds. MSA cases demonstrated an earlier increase in fluorescence but lower maximal fluorescence (Shahnawaz et al., 2020; Singer et al., 2020). The reproducible differences in RT-QuIC reaction kinetics and maximal fluorescence, despite similar quantities of RT-QuIC reaction products implies strain-specific differences in the conformation of RT-QuIC reaction products, which were confirmed with differential binding of amyloid conformation-specific dyes, circular dichroism, Fourier transform infrared spectroscopy, and cryo-electron tomography (Shahnawaz et al., 2020). However, despite these strain-specific differences in the conformation of α-synuclein RT-QuIC reaction products, cryo-electron microscopy data indicates that MSA RT-QuIC reaction products have a different conformation to putaminal MSA α-synuclein seeds (Lövestam et al., 2021). This mirrors the finding in prion disease that RT-QuIC does not recreate infectious prion strains.

### Detection of Co-pathology *In vivo*

There is increasing appreciation of the importance of concomitant proteinopathies in neurodegeneration, with the prevalence of co-pathology ranging from 27% to 68% in different neurodegenerative diseases (Robinson et al., 2018). Taking Alzheimer’s disease as an example, Lewy body and TDP-43 co-pathologies are common and can modify the clinical phenotype and progression. The presence of TDP-43 co-pathology post-mortem is strongly associated with cognitive impairment in elderly patients with AD neuropathologic change, and its absence was associated with normal cognition despite similar degrees of AD pathology suggesting a role in overcoming “cognitive resilience” to AD neuropathology (Josephs et al., 2014; Buciuc et al., 2020). Lewy body co-pathology is associated with more severe executive dysfunction and higher prevalence of hallucinations and poorer scores on the Neuropsychiatric Inventory Questionnaire and Unified Parkinson’s Disease Rating Scale motor scores (Toledo et al., 2013; Chung et al., 2015). In Lewy body disorders, the presence of AD co-pathology is associated with a worse prognosis and shorter interval from motor onset to dementia (Irwin et al., 2017). There is encouraging evidence that CSF α-synuclein RT-QuIC performs similarly in the detection of primary Lewy Body pathology (8/8 positive) and Lewy Body co-pathology in AD, CJD, and primary age-related tauopathy (18/20 positive), with no significant difference in lag phase or peak ThT fluorescence, arguing for a role in the detection of co-pathology and against cross-seeding or inhibition by other aggregating proteins interfering with identification of α-synuclein pathology (Bongianni et al., 2019). Furthermore, a recent study reported detection of α-synuclein RT-QuIC seeding in 3/6 nasal brushing samples and 6/6 CSF samples from mixed DLB/AD (meeting diagnostic criteria for dementia with Lewy bodies but with positive AD biomarkers in CSF), the first time concomitant α-synuclein pathology has been identified by α-synuclein RT-QuIC of nasal brushings (Perra et al., 2021).

Long term cohort studies with proteinopathies detected *in vivo* using a panel of seed amplification assays and confirmed on post-mortem neuropathological examination are warranted and could help to better define the association of co-pathologies with disease phenotype and progression. This should help to stratify clusters of patients according to underlying proteinopathies for inclusion in clinical trials. Furthermore, it could provide a logical basis for inclusion in trials of combination therapy targeting different proteinopathies and eventually contribute to a personalised medicine approach to treating neurodegeneration.

### Application to Alternative and More Accessible Samples

Seed amplification assays have been applied to alternative, more accessible samples to test for proteopathic seeds. This is of particular importance when pre-symptomatic diagnosis and therapeutic intervention are considered, since less invasive methods than CSF sampling are desirable for screening large numbers of asymptomatic people or for serial sampling to measure treatment response. RT-QuIC is so far not applicable to blood, due to inhibition of the RT-QuIC readout, probably because of quenching of ThT fluorescence by the haem moiety (Foutz et al., 2017; Fiorini et al., 2020). PMCA has been successfully applied to blood (in symptomatic and pre-symptomatic patients) and urine, but only for vCJD prions (Moda et al., 2014; Bougard et al., 2016; Concha-Marambio et al., 2016).

In sporadic CJD, RT-QuIC testing of nasal brushings has demonstrated superior sensitivity to CSF RT-QuIC and a combination of RT-QuIC from nasal brushings and CSF has been shown to achieve diagnostic accuracy approaching 100% (Orrú et al., 2014; Bongianni et al., 2017; Fiorini et al., 2020). Orrú et al. (2014) published the first study demonstrating positive RT-QuIC seeding in nasal brushings in 30/31 CJD patients, compared to 23/30 in CSF, indicating greater sensitivity. RT-QuIC responses from nasal brushings were more rapid, exhibited more intense ThT fluorescence and SD_50_ analysis revealed several log_10_ higher seeding capacity in nasal brushings compared to CSF (Orrú et al., 2014). Subsequent studies found near-perfect diagnostic accuracy when RT-QuIC on nasal brushings and CSF were combined. A 100% sensitivity and specificity was reported for sCJD samples from a positive result in CSF or nasal brushing in 61 sCJD patients and on 67 nasal brushing samples and 71 CSF samples from non-prion disease patients (Bongianni et al., 2017). A 100% sensitivity and specificity was reported for positive CSF or nasal brushing RT-QuIC in a prospective study of 182 suspected CJD patients (102 with a final diagnosis of definite or probable CJD, 80 non-prion) all of whom underwent CSF analysis and 42 of whom underwent nasal brushing analysis (Fiorini et al., 2020). This study did not, however, replicate the superior sensitivity of nasal brushing RT-QuIC (91%) over CSF RT-QuIC (96%; Fiorini et al., 2020). A limitation of all of these studies when considering future applications for pre-symptomatic or prodromal disease is that they were undertaken on symptomatic patients suspected to have CJD.

RT-QuIC has also detected prion seeding activity in the eyes and skin of CJD patients; (Orrú et al., 2017, 2018) of these skin biopsy is a more immediately attractive option for an accessible peripheral sample. In skin, initial work was on 35 post-mortem samples and three intra-vitam biopsy samples, in which following a purification step 23/23 CJD patient samples seeded positive RT-QuIC responses in at least one region biopsied and 15/15 non-prion patients did not (Orrú et al., 2017). Sensitivity varied at different biopsy sites: lower back 92%, apex 88%, and around the ear 94% (Orrú et al., 2017). Subsequent studies have replicated high sensitivity and specificity, even without the labour-intensive purification step and including a greater number of samples from living patients (Mammana et al., 2020; Xiao et al., 2021). Mammana et al. (2020) studied biopsy samples from cervical C7 dermatome and lateral thigh of 35 patients with Creutzfeldt-Jakob disease (CJD), including five biopsied intra-vitam, and 36 non-CJD patients and reported 89% sensitivity and 100% specificity. Interestingly there was a preliminary suggestion from this study that seeding capacity in the skin might increase with disease duration, and be lower in sCJD MM1 cases (as the proportion of positive results was lower in these samples, although these do also have a shorter median disease duration and numbers of non-MM1 sCJD cases were low) and also a tentative suggestion of outward spread of seeding capacity from sites closer to the CNS (cervical) to more distant (thigh): in a sCJD MM1 patient, samples taken at 1 month after clinical onset gave a positive response in the cervical region (in 3/4 wells) but not thigh, whereas a post-mortem sample taken 36 months later was fully positive in both locations and with significantly higher fluorescence response and shorter lag phase (Mammana et al., 2020). Similarly, an sCJD MV2K patient 11.5 months after onset had a positive response (in 3/4 wells) only in the cervical region whereas post-mortem both cervical and thigh specimens were fully positive, with a shorter lag phase in the cervical specimen (Mammana et al., 2020). Further studies, with living patients and long-term follow-up with systematic collection of biopsies from different sites are needed to test these preliminary suggestions. Xiao et al. (2021) tested skin biopsy samples at various sites from 51 living patients, 34 clinically diagnosed with probable CJD, three genetic CJD and 14 non-prion cases, and reported sensitivity of skin RT-QuIC as 91% and specificity of 85.7%, although this may need to be interpreted cautiously as concurrent CSF RT-QuIC yielded an unexpectedly low sensitivity (45%).

In α-synucleinopathies RT-QuIC on nasal brushings, skin, salivary gland, and saliva also shows promising sensitivity and specificity in established disease in some recent studies (Manne et al., 2020a; Wang et al., 2020; Perra et al., 2021). De Luca and colleagues reported the first application of α-synuclein RT-QuIC to a more accessible sample than CSF: nasal brushings, which seeded positive responses in 10/18 PD samples, 9/11 MSA samples, and only 1/6 CBD samples and 2/12 PSP samples (De Luca et al., 2019). Subsequent work established reproducibility of a modified version of the same assay between laboratories with 96% inter-rater agreement of results with good sensitivity for MSA with predominant parkinsonism (18/20 results positive, giving a sensitivity of 90%) but a somewhat lower sensitivity for PD (9/13 results positive, giving a sensitivity of 69%) and interestingly only 1/20 positive results in MSA with predominant cerebellar ataxia, which could potentially be attributable to differences in α-synuclein strain, such as tissue tropism (Bargar et al., 2021a). Only 1/22 healthy controls seeded a positive reaction indicating a specificity of 95% (Bargar et al., 2021a). Stefani et al. (2021) assessed α-synuclein RT-QuIC of nasal brushings as a test for prodromal α-synucleinopathy and reported a sensitivity of 44.4% for iRBD, unfavourable when compared to CSF studies (Iranzo et al., 2021) but also reported a sensitivity of 46.3% for Parkinson’s disease (Stefani et al., 2021), which is lower than reported in previous studies on nasal brushings (De Luca et al., 2019; Bargar et al., 2021a), although specificity was high (89.8%). Perra et al. (2021) reported application of α-synuclein RT-QuIC to nasal brushings in 32 probable DLB, five prodromal DLB and six mixed AD/DLB compared with 38 non-α-synuclein neurological disease controls including CJD, AD, PSP, FTD, CBS, and other non-degenerative disorders. In this study, overall sensitivity for DLB was 86.4% and specificity 92.1% (Perra et al., 2021), higher than previously reported for other α-synucleinopathies (De Luca et al., 2019; Bargar et al., 2021a). Interestingly in prodromal DLB 5/5 nasal brushing samples seeded positive reactions (Perra et al., 2021), which seems more promising than the Stefani et al. (2021) study on iRBD, albeit with more limited numbers and a different prodromal phenotype. Very recently, RT-QuIC on saliva has demonstrated 76% sensitivity in distinguishing clinically diagnosed PD patients (57/75 positive) and 61.1% sensitivity in distinguishing clinically diagnosed MSA patients (11/18 positive) from non-neurodegenerative healthy controls, with 94.4% specificity (34/36 controls testing negative; Luan et al., 2022). In all of these studies, relatively low numbers and a lack of neuropathological confirmation of clinical diagnosis are limitations. Further work is required to reproduce findings from single studies, to optimise sensitivity, particularly in prodromal and pre-symptomatic phases, and to understand the dynamics of seeding activity in different tissues in pre-symptomatic and symptomatic phases, requiring long-term follow-up and ideally neuropathological confirmation of diagnosis.

Skin α-synuclein RT-QuIC was reported first by Wang et al. (2020) with sensitivity for PD vs. non-neurodegenerative controls being 94% (44/47) and specificity 98% (42/43) in post-mortem abdominal skin samples. 57 post-mortem α-synucleinopathy patients, including PD, DLB and MSA, and 73 controls were tested including AD, PSP, CBD and non-neurodegenerative controls, sensitivity and specificity were both 93%. When posterior cervical and leg skin biopsy tissues from 20 living patients with PD and 21 controls without PD were tested the sensitivity was 95% and specificity 100% (Wang et al., 2020). Subsequent studies have largely replicated the good sensitivity and specificity of skin α-synuclein RT-QuIC, including sensitivity of 89.2% and specificity of 96.3% in distinguishing 37 Lewy body disease cases from 81 neurological controls in a part clinically diagnosed, part neuropathologically confirmed cohort; (Mammana et al., 2021) sensitivity of 86% and specificity of 80% in 31 living α-synucleinopathy patients, 38 non-synucleinopathy patients and 24 controls (without neuropathological confirmation; Donadio et al., 2021); and sensitivity and specificity of 96% in post-mortem samples from 25 neuropathologically-confirmed PD patients and 25 controls (Manne et al., 2020b). A two-centre comparison study demonstrates sensitivity of 81.8%–90.0% and specificity of 86.7%–90% with 92.2% inter-rater agreement, which is encouraging, but confirmation with greater numbers is warranted given that this included only 34 clinically diagnosed PD cases and 30 controls (Kuzkina et al., 2021).

Application of α-synuclein RT-QuIC to submandibular gland tissue homogenate, in a post-mortem setting, achieved perfect sensitivity and specificity of 94%, in 13 autopsy-confirmed Parkinson’s disease patients, three incidental Lewy body disease patients and 16 controls (Manne et al., 2020a). Whilst this study benefits from neuropathological confirmation, further studies with greater numbers are warranted to establish diagnostic accuracy on antemortem samples.

### Pharmacodynamic Biomarkers

Seed amplification assays provide an attractive way to assess the impact of therapies on proteopathic seeding activity, enabling assessment *in vivo*, rather than on post-mortem histological specimens. This could provide a useful measure of target engagement and could assist in dose-finding in early phase clinical trials, or be used as a surrogate endpoint in later phase studies. The importance of biomarkers as surrogate endpoints is demonstrated by the use of positive biomarker data to facilitate the recent accelerated approval of aducanumab for AD by the US Food and Drug Administration (FDA, 2021). The use of surrogate endpoints can shorten the process of receiving approval since biomarker changes may presage clinical changes and this concept will be particularly important in trials of pre-symptomatic patients. In future clinical practice, quantification of proteopathic seeding activity could be useful for monitoring treatment effectiveness and to detect treatment failure, for example, due to the emergence of drug-resistant prion strains, akin to the use of tumour markers to monitor treatment efficacy in oncology and guide decisions to alter treatment regimens.

Two broad principles have been used to enable the amount of proteopathic seed to be quantified using RT-QuIC: quantitative RT-QuIC (Shi et al., 2013) and endpoint dilution to estimate the seeding activity in terms of SD_50_, the seeding dose giving positive RT-QuIC responses in 50% of replicate reactions (Wilham et al., 2010). Quantitative RT-QuIC utilises dilutions of known quantities of proteopathic seed and measurement of lag time to the exponential rise in ThT fluorescence detected by the RT-QuIC to construct a calibration curve from which the initial quantity of proteopathic seed in the sample of interest can be inferred (Shi et al., 2013, 2015). This method relies heavily on the relationship between quantity of seed and lag time, with the inherent risk of error if other factors are influencing lag time, but requires a smaller volume sample than end-point dilution making it an attractive option if sample volumes are small or the sample particularly scarce (Shi et al., 2013, 2015). The endpoint dilution method relies on serial dilutions with replicate reactions at each dilution to ascertain SD_50_ by Spearman-Kärber analysis, one limitation of which is that a threshold ThT fluorescence must be determined for which RT-QuIC is deemed positive within the time scale of the experiment, which may be somewhat arbitrary. However, such indeterminate RT-QuIC responses are reported to be sufficiently rare in prion disease so as to not significantly impact calculated SD_50_ (Wilham et al., 2010). Potential limitations in both include assumptions regarding seed/substrate compatibility, batch-to-batch variability in reagents and potential for other inhibitors/competitors to be present in the analyte.

When considering quantification of seeding activity as a pharmacodynamic biomarker, inhibition of the RT-QuIC assay due to direct inhibition of *in vitro* seeding by anti-aggregation agents complicates interpretation, although estimation of drug concentration in the sample and comparing its inhibitory effect on the RT-QuIC of samples with known seeding activity may aid interpretation (Ding et al., 2021). Another complexity of interpretation of RT-QuIC in trials of anti-aggregation agents to consider, particularly in the context of treatment failure, is whether the emergence of a drug-resistant protein strain would be detectable or not, due to potential alteration in RT-QuIC amyloid seeding activity. These potential complexities are not anticipated in therapeutics with other mechanisms such as genetic therapies aiming to deplete protein of interest by reducing expression, or therapies enhancing the clearance of misfolded proteins (Abdelaziz et al., 2019; Minikel et al., 2020).

A potential limitation is the patient tolerability of serial lumbar puncture during the clinical trial, although seed amplification assays have been applied to other more accessible analytes. Interestingly in a mouse model of prion disease skin RT-QuIC seeding activity was abolished by treatment with an anti-prion agent that prolonged survival, suggesting a potential role for skin RT-QuIC as a pharmacodynamic biomarker (Ding et al., 2021). However, despite the ease of skin biopsy relative to lumbar puncture, CSF remains a more attractive analyte for assessing drug target engagement in the central nervous system owing to its proximity to the brain parenchyma. Clinical experience in HIV treatment, where uncontrolled HIV viral load may be present in CSF despite undetectable levels in blood highlights the risk of over-reliance on peripheral biomarkers (Rawson et al., 2012).

## Expansion into Tauopathies

Tauopathies underlie a broad range of human neurodegenerative diseases, which share accumulation and deposition of tau aggregates as a common feature. Tau pathology may be the primary pathology, as in Pick’s disease, progressive supranuclear palsy, and corticobasal degeneration, or a secondary pathology as in Alzheimer’s disease or certain forms of prion disease. As a result of alternative splicing adults normally express tau with either three or four microtubule binding repeat domains (3 or 4R), and different tauopathies have aggregates with differing isoform usage, in keeping with different tau fibril structural strains underlying different diseases (Stahlberg and Riek, 2021). RT-QuIC has been adapted to tauopathies of 3R, 4R, and 3R/4R tau isoforms, which with further testing could be a valuable tool for differential diagnosis of tauopathies.

Saijo et al. (2017) reported the first tau RT-QuIC, which utilises K19CFh synthetic 3R tau fragment as substrate and was developed using Pick’s disease brain homogenates but attained 91%–100% sensitivity when applied to post-mortem Pick’s disease CSF and 94%–100% specificity when applied to ante-mortem control CSF. Other studies detecting 3R tau seeds have used brain homogenate samples only (Kraus et al., 2019; Metrick et al., 2020; Tennant et al., 2020). The addition of a second 4R tau substrate (τ306) extended the application of RT-QuIC to detect 3R and 4R tau deposits in brain homogenate from AD and CTE (Kraus et al., 2019). The development of RT-QuIC with tau K12CFh synthetic tau fragment enabled one assay to detect and discriminate (by differing ThT fluorescence maxima) 3R seeds of Pick’s disease and 3R/4R seeds of CTE and AD brain homogenate (Metrick et al., 2020). 4R tau RT-QuIC has also been developed, and yielded promising data from postmortem CSF in which 4/4 CBD and 7/7 PSP cases were positive (≥50% of the replicate reactions above the fluorescence threshold) and 2/2 controls tested negative (Saijo et al., 2020). In antemortem CSF the seeding activities were weaker but mean responses from clinically diagnosed PSP and CBS were significantly higher than controls (including healthy controls and neurological controls thought not to have 4R tauopathy; Saijo et al., 2020). Application of these techniques to CSF or more accessible samples from patients to provide accurate intra-vitam biochemical confirmation of tauopathy diagnosis, and quantification of seeding activity, requires further work, but would be important since tauopathies underlie some of the most common neurodegenerative disorders including AD and tau is a promising drug target for clinical trials.

## Expansion into TDP-43 Proteinopathies

TDP-43 aggregates are present in 97% of amyotrophic lateral sclerosis and around 45% of frontotemporal lobar degeneration cases and substantial evidence has accrued that TDP-43 exhibits prion-like properties of seeded aggregation and cell-to-cell spreading (Ling et al., 2013). Scialò et al. (2020) reported the first RT-QuIC adapted to this proteinopathy in 2020. They used synthetic human TDP-43 seeds to establish C-terminal fragment human TDP-43 (263-414) as an efficient substrate displaying typical RT-QuIC kinetics when seeded with synthetic seed, with higher ThT fluorescence and a shorter lag phase when compared to RT-QuIC reactions utilising full-length human TDP-43. Atomic force microscopy revealed differences in the products of these reactions with amorphous aggregates produced from full-length TDP-43 substrate and fibrillar aggregates from the TDP-43 (263–414), which may explain the favourable RT-QuIC readout using TDP-43 (263–414; Scialò et al., 2020).

The assay was further optimised using TDP-43 proteinopathy patient brain homogenate and then adapted to CSF from patients (and one pre-symptomatic carrier) with *C9ORF72* hexanucleotide repeat expansions, *GRN* mutations, and *TARDBP* mutations, expected to have underlying TDP-43 pathology. The addition of CSF inhibited TDP-43 (263–424) aggregation somewhat and CSF samples provided rather noisy RT-QuIC readouts. Nonetheless using 36 patient CSF samples and 27 age-matched controls free from neurodegenerative disorders, patients and controls could be discriminated with sensitivity of 94% and specificity of 85%. This specificity falls below that achieved by RT-QuIC in prion disease and further optimisation of the assay is warranted. Of the controls who were RT-QuIC positive, two were re-tested following immune-depletion of TDP-43 and one remained positive, implying that at least some of the false positives were genuine false positives (rather than detection of TDP-43 seeds in pre-symptomatic individuals; Scialò et al., 2020). The authors suggest potential unknown factors in patient CSF altering RT-QuIC reaction kinetics, which might be removed by a sample preparation step, but it is also plausible that further optimisation of the assay for CSF samples could be achieved by iterative changes in combinations of temperature, shaking parameters, the composition of reaction mix, and the choice of substrate as has been done in RT-QuIC for prion disease (Orrú et al., 2016; Metrick et al., 2019). Interestingly, the one pre-symptomatic *GRN* mutation carrier was TDP-43 RT-QuIC positive, suggesting potential for TDP-43 RT-QuIC in pre-symptomatic diagnosis. Further studies should include serial samples from greater numbers of patients, including pre-symptomatic mutation carriers and patients with sporadic FTD and ALS, and long term follow-up to enable post-mortem neuropathological confirmation of TDP-43 neuropathology and to provide the opportunity to identify phenoconversion from pre-symptomatic TDP-43 proteinopathy. Another area for development would be the quantification of seeding activity, which was not possible in this study (Scialò et al., 2020).

## Expansion into Inherited Prion Diseases

The development of effective RT-QuIC assays in a diverse range of inherited prion diseases (IPDs) is of great interest in the context of future clinical trials of disease-modifying therapeutics targeting PrP in asymptomatic mutation carriers. RT-QuIC could represent a pre-symptomatic disease activity biomarker indicating that the formation of PrP proteopathic seeds is underway. This would provide a rational entry criterion for starting treatment in mutation carriers and measurement of seeding activity with RT-QuIC (or “switching off” of a binary RT-QuIC readout) could measure the effect of treatment on depleting PrP proteopathic seeds. Furthermore, the exquisite sensitivity of RT-QuIC, and the proposed 2-phase model of prion kinetics, in which a rapid increase in infectious prion titre is followed by a plateau in prion titre during which toxic species accumulate (Sandberg et al., 2011, 2014), suggests that such a change could be detected prior to substantial neuronal damage-causing changes in secondary biomarkers of neuronal damage such as neurofilament light chain, which is the most promising biomarker for proximity to phenoconversion in IPDs at present (Thompson et al., 2021).

Second-generation “IQ-CSF” RT-QuIC has demonstrated good performance in IPDs with a CJD phenotype also known as genetic CJD (gCJD), such as the E200K and V210I *PRNP* mutations, with sensitivities as high as 100% reported and there is preliminary data showing promise for olfactory mucosa RT-QuIC as well (Bongianni et al., 2017; Foutz et al., 2017; Franceschini et al., 2017; Lattanzio et al., 2017). Franceschini et al. (2017) reported 100% sensitivity with second generation RT-QuIC with positive RT-QuIC in 20/20 E200K cases, 10/10 V210I cases, 1/1 D178N-CJD case, 1/1 R208H case, and 1/1 E219G case. Lattanzio et al. (2017) reported 20/20 positive results in E200K, 20/21 in V210I, 1/1 in 4-OPRI, 0/2 in D178N-CJD, 0/1 in R208H and 1/1 in V203I. Foutz et al. (2017) also reported positive RT-QuIC in 14/14 gCJD cases, and Bongianni reported 5/6 gCJD cases positive on either first or second generation RT-QuIC and 5/6 cases positive using the olfactory mucosa RT-QuIC (Bongianni et al., 2017).

RT-QuIC performance in identifying slowly progressive inherited prion diseases including Gerstmann-Straussler-Scheinker syndrome IPDs and in fatal familial insomnia caused by D178N *PRNP* mutation appears less good. For example, Franceschini et al. (2017) found positive RT-QuIC seeding in 1/4 P102L cases, 0/2 D178N-FFI cases, 1/1 A117V cases, 0/1 D202N cases, and 0/1 octapeptide repeat insertion (OPRI) cases; Foutz et al. (2017) found positive RT-QuIC seeding in 1/2 P102L cases and 1/1 D178N cases; and Bongianni et al. (2017) reported RT-QuIC negative result in a single P102L case using first-generation RT-QuIC but 1/2 P102L cases positive using olfactory mucosa RT-QuIC. There is only one published report of RT-QuIC in non-CJD phenotype IPDs yielding similar sensitivity to sCJD, in which full-length human rPrP was used as a substrate, which may explain their success where other RT-QuIC methods have failed (Sano et al., 2013). In this study by Sano et al. (2013), seeding activity was detected in CSF of 18/22 E200K, 18/20 P102L, and 10/12 D178N-FFI cases.

Bank vole PrP has been proposed as a universal acceptor of prions and a universal substrate for prion strains in RT-QuIC (Orrú et al., 2015b). Recent work on post-mortem CSF has broadened the use of recombinant bank vole PrP RT-QuIC to detect prions in slow IPDs including 6-OPRI (Mok et al., 2021). However, several studies produced results at variance with the proposed status of bank vole PrP as a universal RT-QuIC substrate with significantly lower sensitivities (52.6%–88.6%) than achieved with IQ-CSF RT-QuIC, which uses truncated hamster PrP (Vallabh et al., 2020; Mok et al., 2021). Work from animal bioassays in P102L and A117V slowly progressive IPDs indicate that efficient prion transmission only occurs in transgenic mice expressing human PrP with the same mutation (Asante et al., 2013, 2015); similarly for some IPDs mutant rPrP may be required as an optimal RT-QuIC substrate. Further work is required to establish the optimal RT-QuIC substrates and conditions to detect seeding activity in the non-CJD phenotype IPDs including slowly progressive IPDs and fatal familial insomnia. Longitudinal studies will be vital in establishing changes in RT-QuIC seeding activity in well-defined cohorts of IPD before and after phenoconversion to enable its use as a biomarker of disease activity and to facilitate potential use in clinical trials. International collaboration may be required to accrue sufficient numbers in rare forms of IPD.

## Expansion into Inherited Parkinson’s Disease

The RT-QuIC assay is accruing very encouraging data in a range of α-synucleinopathies, with multiple studies indicating sensitivities and specificities in excess of 90%, including in pathologically defined cohorts (Bargar et al., 2021b). One area for future development is its role in inherited forms of Parkinson’s disease where cohorts of mutation carriers, similarly to inherited prion disease, provide an outstanding opportunity for research. The figures of 40% sensitivity and 80% specificity (the lowest of published studies of alpha-synuclein RT-QuIC) from a study of *LRRK2*-associated Parkinson’s disease might appear discouraging (Garrido et al., 2019). However, this may be due to non-synuclein pathology in some *LRRK2*-associated PD, which is known to have a variable neuropathological basis or differences in the seeding propensity of *LRRK2*-associated α-synuclein strains with currently used substrates (Garrido et al., 2019). Interestingly, some non-symptomatic mutation carriers exhibited RT-QuIC seeding activity and a higher proportion of those that did fulfilled Movement Disorder Society criteria for prodromal PD (Garrido et al., 2019). This highlights the potential of α-synuclein RT-QuIC as a marker of pre-symptomatic/prodromal α-synucleinopathy (with possible contribution to patient selection in clinical trials) once established in greater numbers of mutation carriers and with longitudinal studies, ideally with post-mortem histopathological confirmation (Garrido et al., 2019). A pre-symptomatic marker of α-synucleinopathy that predicts disease would be of particular relevance to pre-symptomatic treatment in *LRRK2* mutation carriers since penetrance is incomplete with estimates of penetrance of the most common *LRRK2* p.G2019S mutation ranging from 25% to 42.5% (Lee et al., 2017).

In light of the variable neuropathological basis of genetic forms of PD, α-synuclein RT-QuIC has been applied to a cohort enriched with genetic forms of PD and DLB, including 115 *GBA* mutations, which tend to be associated with extensive α-synuclein-positive Lewy body pathology, 20 autosomal recessive PD mutations (*PRKN, PINK1, and DJ1*), associated with nigral degeneration in the absence of Lewy bodies and nine *LRRK2* mutations, associated most often with Lewy body pathology, but also tau pathology and nigral degeneration in the absence of Lewy bodies (Brockmann et al., 2021). Reflecting the expected underlying neuropathology, symptomatic patients with *GBA* mutations, particularly those classed as severe, demonstrated RT-QuIC seeding activity most frequently (93% in PD, 100% in DLB), whereas those with homozygous autosomal recessive PD mutations did not seed any RT-QuIC responses and those with *LRRK2* mutations demonstrated an intermediate frequency of seeding activity (78%; Brockmann et al., 2021). The overall proportion of pre-symptomatic mutation carriers (1 *GBA*, 1 *LRRK2*) that tested positive was low (14%) and similar to controls (8%); further longitudinal studies are warranted to investigate changes in seeding activity over time, particularly in patients that phenoconvert, in order to determine the clinical utility of RT-QuIC seeding as a biomarker for proximity to phenoconversion (Brockmann et al., 2021).

## Expansion into Huntington’s Disease

Huntington’s disease is the most common monogenic neurodegenerative disease amongst populations of European ancestry and causes behavioural symptoms, progressive movement disorder, and cognitive decline with a variable age of onset (Kay et al., 2018). It also presents an opportunity to develop biomarkers and treatments for use in pre-symptomatic and early symptomatic disease thanks to a significant number of asymptomatic individuals who are aware of their at-risk or mutation carrier status. In Huntington’s disease, a CAG repeat expansion in exon 1 of the *HTT* gene results in the production of mutant huntingtin (mHTT) with an expanded polyglutamine stretch. The length of polyglutamine expansion is inversely related to time to motor onset (Lee et al., 2012), and longer polyglutamine expansions are associated with a greater propensity for aggregation (Li and Li, 1998). Ultrasensitive immunoassays have been used to measure mHTT in patient CSF and also used as readouts to provide evidence of genetic therapies’ depletion of mHTT (Wild et al., 2015; Tabrizi et al., 2019). However these assays do not attempt to measure the functional property of aggregation seeding. Seed amplification assays have the potential to quantify aggregation-prone HTT seeds in CSF of patients with Hungtington’s disease or those carrying a pathogenic HTT mutation, and given the exquisite sensitivity afforded by such assays in other protein misfolding disorders might potentially be applicable as biomarkers of disease onset and disease activity.

No cell-free amplification assays, such as RT-QuIC or PMCA, have yet been published for biofluids in patients with Huntington’s disease, although cell-based aggregation assays revealed that synthetic polyglutamine oligomers and HD patient CSF seeded aggregation and, intriguingly, healthy pre-symptomatic HD mutation carriers displayed a range of seeding activities spanning healthy controls and HD patients, suggesting the possibility of seeding activity as a biomarker of disease progression, even in pre-symptomatic stages (Tan et al., 2015). A different cell-based seeding assay shows promise with detection of seeding activity in aggregates of mutant HTT and HD patient CSF, and significant elevation of seeding activity with disease onset and progression of neuropathological grades, although no increase in prodromal disease (Lee et al., 2020). Although not seed amplification assays, several immunoassays have been developed to detect aggregated huntingtin (HTT) in mouse models of HD, including mesoscale discovery (MSD) assays and TR-FRET-based assays (Baldo et al., 2012; Reindl et al., 2019). Whilst the antibodies used in these assays bind with high specificity to HTT, and with a propensity for aggregated HTT, a significant drawback of these approaches to quantifying HTT is that so far none of the MSD assays has been demonstrated to be sensitive enough to detect aggregated HTT in human samples, which have lower aggregate load than mouse models used in their development. Perhaps in future, a cell-free amplification assay could be developed to provide the scalability, high-throughput capacity, sensitivity and specificity required for a clinical laboratory test for the emergence of aggregation-prone HTT seeds in patient CSF, with the possible applications as a biomarker of proximity to phenoconversion or a pharmacodynamic biomarker in trials of therapy.

## Conclusion

Seed amplification assays, such as RT-QuIC, provide a sensitive and specific means of identifying PrP proteopathic seeds in biofluids from patients with sCJD and have been adopted into the diagnostic criteria (European Centre for Disease Prevention and Control, 2017). Recent advances in other neurodegenerative diseases including α-synucleinopathies, tauopathies, and TDP-43 proteinopathies suggest a similar potential for identifying the proteopathic seeds thought to be central to the pathogenesis of these disorders *in vivo*. Seed amplification assays may have an important role to play in therapeutic intervention by matching therapeutic interventions to the correct underlying pathology and because waiting for the full gamut of clinical signs to appear to meet clinical diagnostic criteria may delay treatment beyond the stage at which it has the highest chance of success. Other possible applications of seed amplification assays to disease classification include studies into the impact of co-pathology on disease phenotype, progression, and response to therapeutics.

In future, a panel of seed amplification assays for different proteopathic seeds might be employed to test specimens from patients with suspected neurodegeneration, or even in pre-symptomatic individuals, to enable intra vitam biochemical confirmation of the underlying pathology. However, further work would be helpful to establish the diagnostic accuracy of these seed amplification assays in large-scale longitudinal studies, ideally with neuropathological verification of assay findings, and to ensure the standardisation and reproducibility between centres, prior to implementation of such a panel.

Quantification of proteopathic seeding activity by seed amplification assays may also provide a measure of disease activity, with the potential to guide when to start pre-symptomatic treatment in protein misfolding disorders and to provide a pharmacodynamic biomarker but again longitudinal studies are required to assess the performance of different seed amplification assays in these roles. Other areas for future development include the detection of other proteins that aggregate in neurodegenerative disorders, such as huntingtin, superoxide dismutase, and fused in sarcoma protein (McAlary et al., 2019), and further adaptation and optimisation of assays utilising accessible biosamples to minimise the invasiveness of intra-vitam diagnosis.

## Author Contributions

TC drafted the manuscript and reviewed the literature. SM conceived the project and edited the manuscript. All authors contributed to the article and approved the submitted version.

## Conflict of Interest

TC is an Association of British Neurologists/Alzheimer’s Research UK clinical research training fellow and is funded by the Association of British Neurologists and Alzheimer’s Research UK. The remaining author declares that the research was conducted in the absence of any commercial or financial relationships that could be construed as a potential conflict of interest.

## Publisher’s Note

All claims expressed in this article are solely those of the authors and do not necessarily represent those of their affiliated organizations, or those of the publisher, the editors and the reviewers. Any product that may be evaluated in this article, or claim that may be made by its manufacturer, is not guaranteed or endorsed by the publisher.
